# Pill to Pain: First Case of Topiramate-Induced Chronic Spontaneous Coronary Artery Dissection (SCAD)

**DOI:** 10.7759/cureus.13263

**Published:** 2021-02-10

**Authors:** Tanvir Rahman, Reihaneh Moghadam, Morton Rinder

**Affiliations:** 1 Internal Medicine, St. Luke's Hospital, Chesterfield, USA; 2 Cardiology, St. Luke's Hospital, Chesterfield, USA

**Keywords:** coronary artery angiography, scad types, scad management, topiramate, chronic migraine, chronic scad

## Abstract

Spontaneous coronary artery dissection (SCAD) is a non-traumatic, non-iatrogenic, and non-atherosclerotic coronary artery disorder that manifests clinically as acute coronary syndrome (ACS), arrhythmia, or sudden cardiac death (SCD). It is a rare cause of ACS (1.7%-4%) and SCD (0.5%), more common in women than men. It was first reported in 1931 in a 42-year-old female at autopsy, who had SCAD after violent retching and vomiting. We report a case of a 51-year-old female who developed sudden-onset chest pain after taking topiramate (TPM). Her chest pain persisted for 1.5 months prior to her outpatient evaluation, which led to further cardiac workup. An urgent left heart catheterization (LHC) revealed a SCAD. Her symptoms improved with percutaneous coronary intervention (PCI), and she was discharged home on aspirin, statins, and beta-blockers.

## Introduction

Spontaneous coronary artery dissection (SCAD) is a non-obstructive coronary artery disease clinically representing as acute coronary syndrome (ACS), arrhythmia, or sudden cardiac death (SCD). SCAD is a rare cause of ACS (1.7%-4%) [1] and SCD (0.5%) [2]. First reported in 1931 in an autopsy of a 42-year-old female who was diagnosed with SCAD following vigorous retching and vomiting [3]. More than 50% of patients recall a precipitating factor, including intense exercise (isometric or aerobic), intense Valsalva, retching, vomiting, bowel movement, coughing, lifting heavy objects, intense emotional stress, labor, and delivery, and recreational drugs (cocaine, methamphetamines), exogenous hormones/hormone modulators β-hCG injections, corticosteroids [4].

The prevalence of SCAD might be higher than previously thought. A recent study showed the occurrence of SCAD in 4% of cases out of 326 patients, who underwent routine optical coherence tomography (OCT) [1]. The mean age of the SCAD population was 44±9 years, and 92% were women with low rates of atherosclerotic risk factors [5]. Recent case series suggest women accounted for over 90% of cases of SCAD [6]. Patients with pregnancy-related SCAD trended toward more often having a prior history of infertility treatment (28% vs. 16%; p = 0.055), including selective estrogen receptor modulators (8 of 15), gonadotropin therapy (5 of 15), and aromatase inhibitors (2 of 15) [7]. During the peripartum period, cocaine-abusing women were highly susceptible to MI caused by the effect of cocaine on a heart that is already stressed by hemodynamic changes of pregnancy. The diagnosis of SCAD should be considered in any postpartum patient who presents with MI, particularly in the setting of cocaine use [8]. The report of SCAD occurring soon after triptan exposure could be due to triptan-induced vasoconstriction in patients with underlying vascular fragility [9].

## Case presentation

The patient is a 51-year-old female who presented to her cardiologist’s office with complaints of sudden-onset chest pain continuing for 1.5 months. The pain was first-time experienced shortly after taking her doubled dose migraine pill (topiramate). The pain was intermittent, a pressure-like sensation associated with diaphoresis, radiation down her left arm at rest, and worsened with any activity. Since the onset, her chest pain occurred on a daily basis including waking her up in the morning from severe pain. She has known gastroesophageal reflux disease (GERD) and called her gastroenterologist but was subsequently referred to the cardiologist’s office to rule out acute coronary syndrome (ACS). The cardiologist sent her to the emergency room (ER) due to the history of resting chest pain and abnormal electrocardiogram (ECG). She was not in pain on presentation to the ER. The patient denied any shortness of breath, nausea, vomiting, or syncope except for some diaphoresis. Her heart rate and blood pressures were 82 bpm and 128/70 mmHg, respectively. No murmur was heard on physical exam, and the lungs were clear to auscultation without any crackles or wheezing.

Past medical history was significant for chronic migraine headaches, fibromyalgia, anxiety disorder, pseudo-seizures, irritable bowel syndrome, gastroesophageal reflux disease (GERD), severe protein-calorie malnutrition, and premature surgical menopause. Her migraines have been so debilitating that she had to go on disability.

Medications included albuterol two puffs every four hours as needed for shortness of breath, baclofen 10 mg twice a day, diazepam 5 mg three times a day as needed, esomeprazole 40 mg twice a day, famotidine 40 mg daily, linaclotide 20 mcg oral daily, metoprolol 12.5 mg twice a day, ondansetron 8 mg three times a day as needed, topiramate 300 mg twice a day (increased from 200 mg twice a day). She is a lifetime non-smoker, non-alcoholic, and never used illicit drugs.

The differential diagnosis included ACS, coronary vasospasm, GERD, and panic attack.

**Figure 1:**
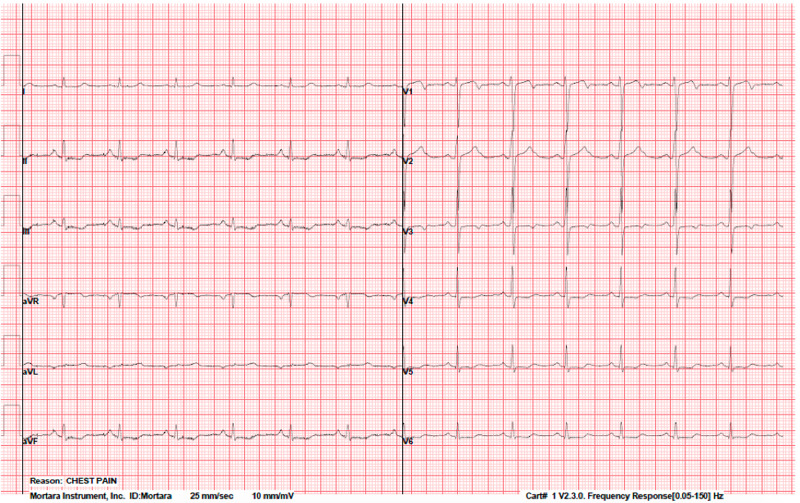
ECG upon presentation to the ER ECG upon presentation to the ER showed mild ST-segment depression at II, III, aVF, and V3-V6. ECG: electrocardiogram; ER: emergency room

ECG upon presentation to the ER was significant for mild ST-segment depression at II, III, aVF, and V3-V6 (Figure 1). Troponins (TnI) were negative and remained negative throughout the admission. She tested negative for severe acute respiratory syndrome coronavirus 2 (SARS-CoV-2).

**Figure 2:**
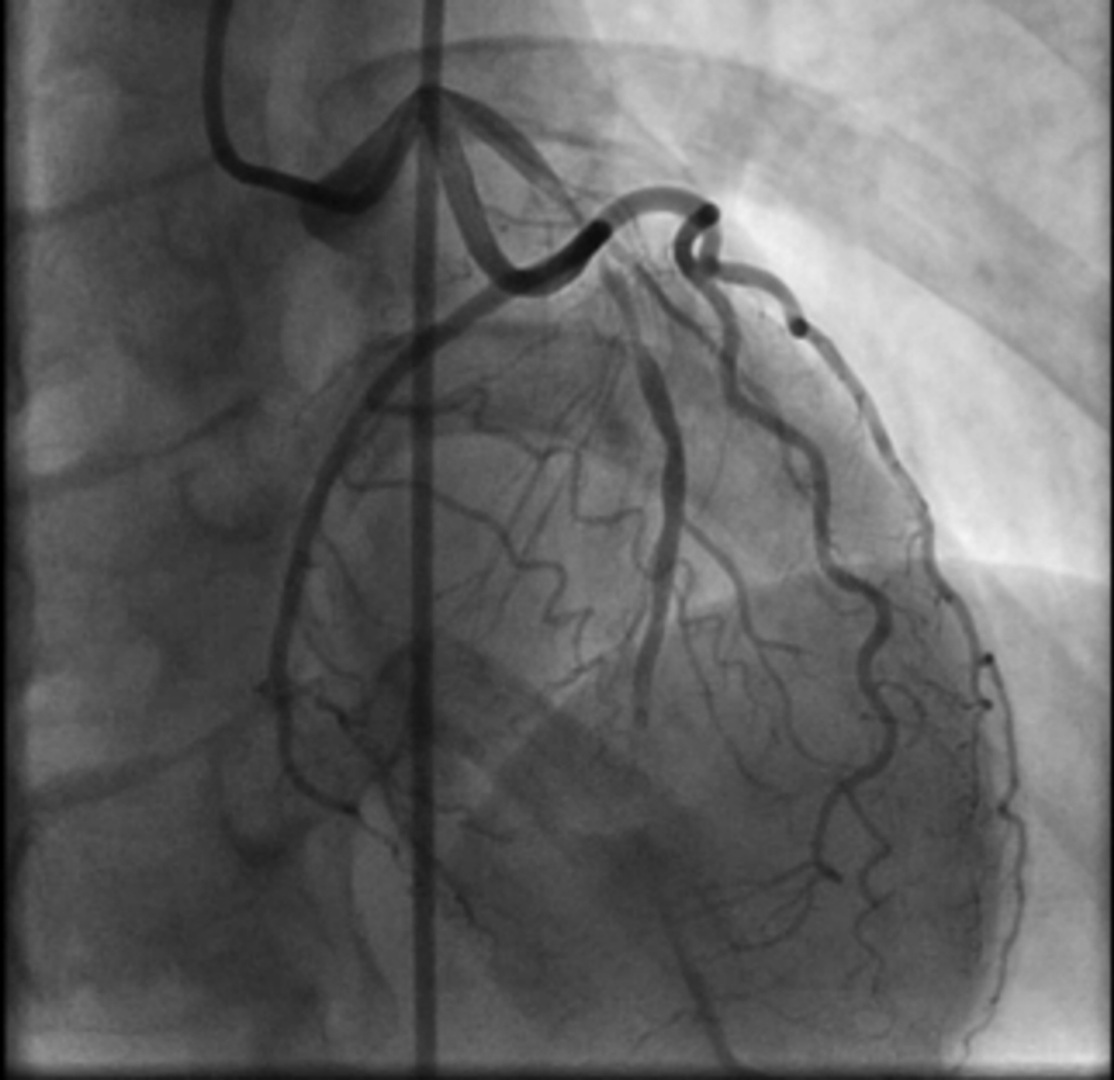
The proximal and mid-portion of the LAD artery contained 99% and 40% stenosis, respectively, with TIMI 1 flow LAD: left anterior descending; TIMI: thrombolysis in myocardial infarction

Given her recurrent chest pain, EKG with ST depressions in the anterior leads and premature surgical menopause, a decision was made to go for left heart catheterization (LHC) after beginning aspirin and metoprolol. She was very reluctant to take any medications and has multiple drug intolerances. The left main coronary artery was free of angiographic evidence of coronary artery disease. The proximal and mid-portion of the LAD artery contained a long 99% and 40% stenosis, respectively, with thrombolysis in myocardial infarction (TIMI) 1 flow (Figure 2).

**Figure 3:**
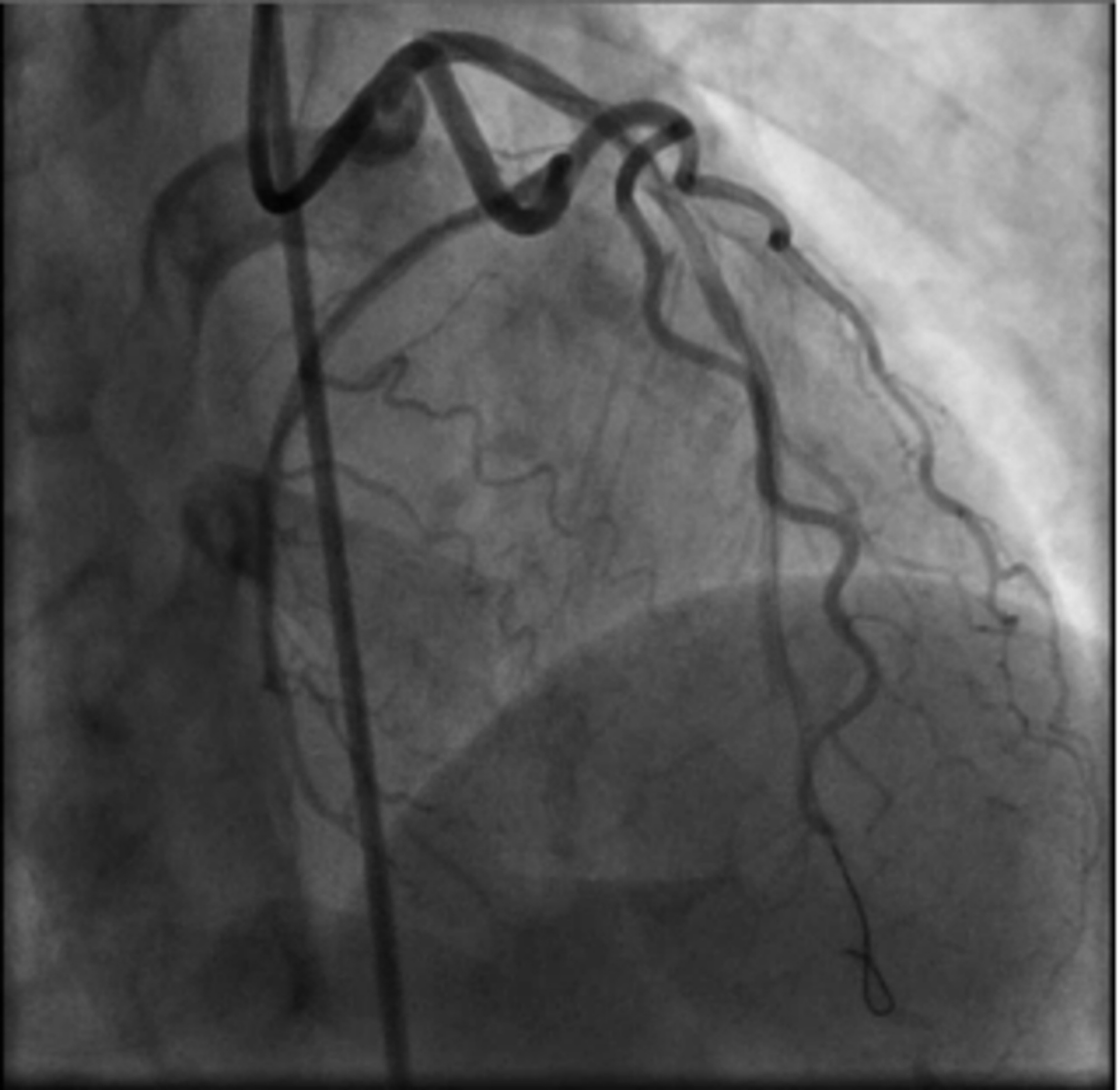
SCAD was successfully crossed with the Luge wire and predilated SCAD: spontaneous coronary artery dissection

The diagonal, circumflex (LCX), obtuse marginal (OM), and right coronary arteries (RCA) did not show any dissection but luminal irregularities throughout the vessel. Left ventricular ejection fraction (LVEF) was measured at 60%. The proximal LAD lesion appeared to be a chronic SCAD with the diminished flow into the mildly diseased mid and distal LAD. This patient with unstable anginal symptoms and a high-grade lesion involving the LAD treated percutaneously with a drug-eluting stent. A successful PCI of the proximal left anterior descending artery was performed using a Luge wire to carefully traverse the dissection and stay in the true lumen (Figure 3).

**Figure 4:**
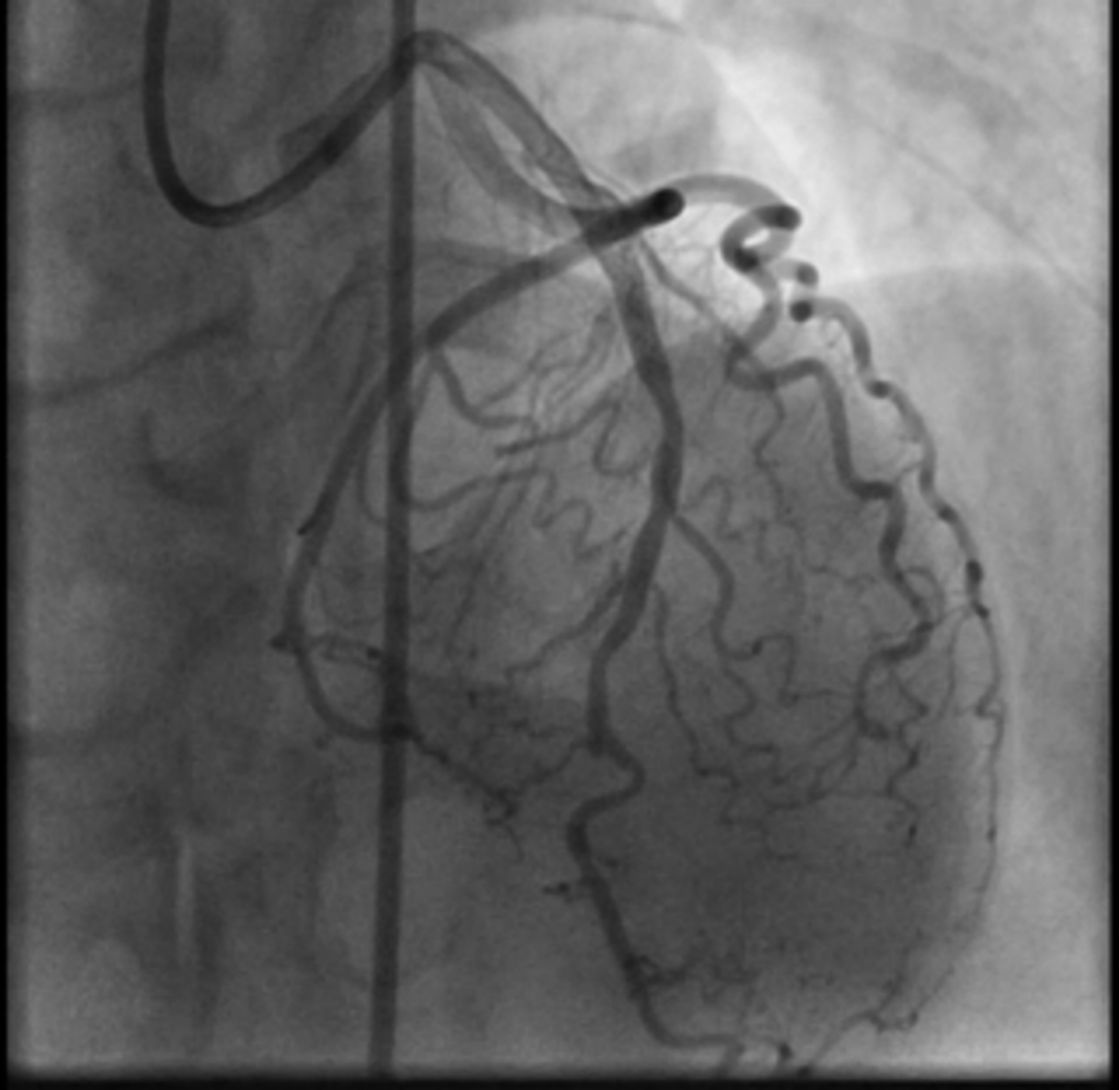
The successful placement of the DES to LAD with post-PCI stenosis of 0% with the restoration of normal TIMI 3 flow DES: drug-eluting stent; LAD: left anterior descending; PCI: percutaneous coronary intervention; TIMI: thrombolysis in myocardial infarction

The lesion was stented and post dilated successfully. Post-PCI stenosis was 0% with TIMI 3 flow (Figure 4).

**Figure 5:**
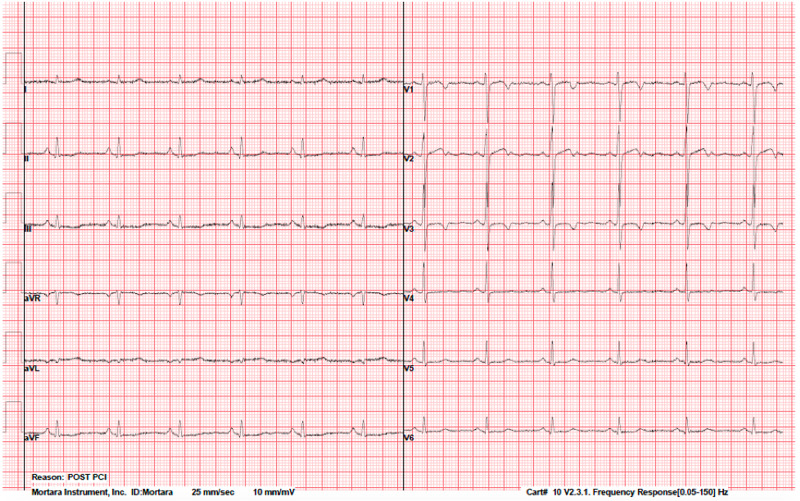
Post-PCI ECG showed ST-segment depressions improvement, new T-wave inversions, and flattening over the precordial leads PCI: percutaneous coronary intervention; ECG: electrocardiogram

The left ventricular end-diastolic pressure and left ventricular ejection fraction were normal. Post-PCI ECG showed mild improvement of ST-segment depressions, new T-wave inversions, and flattening over the precordial leads (Figure 5).

The patient was continued on aspirin 81 mg daily, metoprolol 12.5 mg twice a day, and was placed on ticagrelor 90 mg twice a day and rosuvastatin 10 mg daily.

## Discussion

This is the first reported case to our knowledge of topiramate-induced SCAD. The patient was allergic to sumatriptan and was placed on topiramate with an eventual increase in dose. The patient was already prone to SCAD due to having the diagnosis of fibromyalgia and was a female of reproductive age. The patient had intermittent chest pain for approximately 1.5 months mimicking the presentation as unstable angina yet had a very distinct history of developing the abrupt onset of pain within a short time of taking her migraine medication. The absence of a wall motion abnormality or a troponin elevation despite resting pains suggests that the flow through the true lumen remained adequate enough to avoid infarction of the anterior wall. This type of varying pain is very consistent with our experience with other SCAD patients who develop waxing and waning symptoms, which can, at times, result in ST-segment elevation and then resolve spontaneously with medication therapy. Our patient had type 2 SCAD with a long, diffuse, and smooth narrowing of the proximal and mid-portion of LAD, which ultimately required stent treatment due to ongoing ischemic-like resting chest pains and TIMI 1 flow. There have been reports on triptan-induced SCAD but none on topiramate-induced SCAD to our knowledge [9-10]. Fibromuscular dysplasia (FMD) was found to be a comorbid factor in both case reports. In contrast to the previous cases, our patient was older and had been diagnosed with fibromyalgia, which has never been associated with vasculopathy, though we have not searched for evidence of FMD. Furthermore, our patient did have some evidence of mild CAD on the angiogram, especially in the LAD just beyond the distal edge of the proximal dissection. Ironically, this mild CAD plaque acted as a barrier to the propagation of the dissection plane so that it contained the SCAD to the proximal vessel and may have prevented an acute myocardial infarction.

Topiramate has several mechanisms of action, most commonly known as a voltage-gated sodium channel blocker. FDA approved it in 2004 for migraine prophylaxis but the exact mechanism of action remains unknown [11]. There is scope to find out if there is any association of topiramate with adverse cardiac outcomes.

## Conclusions

In summary, to the best of our knowledge, this is the first reported case of topiramate-induced SCAD. Though we are not aware of the exact mechanism, it is worthwhile to report this case as further research is necessary to rule out any cardiovascular side effects of this medication.
